# Supplementary material for: Metabolic rate in common shrews is unaffected by temperature, leading to lower energetic costs through seasonal size reduction
Source: R Soc Open Sci. 2020 Apr 22;7(4):191989. doi: 10.1098/rsos.191989 (PMC7211839; doi:10.1098/rsos.191989)
Supplement: Table S1. [file rsos191989supp1.docx]

Table S1. All model results evaluating the effects of body mass, standardized skull height (BCH), temperature, and season on absolute metabolic rates (VO2 ml min^-1^) and relative metabolic rates (VO2 ml kg^-1^ min^-1^) and include individual ID as a random intercept, season as a random slope, and continuous time autocorrelation structure. Body mass, skull height, and temperature were all scaled prior to analysis. Models are shown in order of their AICc rank.

| **Total MR**  **(VO2 ml min^-1^) ~** | | **AICc** | **Factors** | | **Estimate ± SE** | | **t-value [DF]** | | **p-value** | | **Anova LME F [DF]** | | **p-value** | |  |
| --- | --- | --- | --- | --- | --- | --- | --- | --- | --- | --- | --- | --- | --- | --- | --- |
| mass | | -34226.92 | (Intercept) | | 1.42 ± 0.03 | | 45.17 [13262] | |  | |  | |  | |  |
|  | |  | mass | | 0.42 ± 0.03 | | 13.38 [18] | | >0.001 | | mass: 179.07 [1,18] | | <0.001 | |  |
| season | | -34216.73 | (Intercept) | | 1.31 ± 0.06 | | 21.85 [13262] | | 0.001 | | season: 76.67 [2,17] | | <0.001 | |  |
|  | |  | season (winter) | | -0.25 ± 0.08 | | -3.1 [17] | | 0.01 | |  | |  | |  |
|  | |  | season (spring) | | 0.75 ± 0.09 | | 8.6 [17] | | >0.001 | |  | |  | |  |
| mass*season | | -34215.36 | (Intercept) | | 1.48 ± 0.12 | | 11.88 [13262] | |  | |  | |  | |  |
|  | |  | mass | | 0.47 ± 0.31 | | 1.51 [14] | | 0.15 | | mass: 171.94 [1,14] | | <0.001 | |  |
|  | |  | season (winter) | | -0.14 ± 0.26 | | -0.56 [14] | | 0.59 | | season: 0.5 [2,14] | | 0.62 | |  |
|  | |  | season (spring) | | 0.07 ± 0.29 | | 0.23 [14] | | 0.82 | |  | |  | |  |
|  | |  | mass*season (winter) | | -0.12 ± 0.42 | | -0.29 [14] | | 0.78 | | mass*season: 0.07 [2,14] | | 0.93 | |  |
|  | |  | mass*season (spring) | | -0.13 ± 0.35 | | -0.36 [14] | | 0.72 | |  | |  | |  |
| mass*temperature | | -33166.28 | (Intercept) | | 153.19 ± 4.39 | | 34.91 [12826] | |  | |  | |  | |  |
|  | |  | mass | | -1.8 ± 4.34 | | -0.41 [18] | | 0.68 | | mass: 168.11 [1,18] | | <0.001 | |  |
|  | |  | temperature | | 1.06 ± 3.98 | | 0.27 [12826] | | 0.79 | | temperature: 0.05 [1,12826] | | 0.82 | |  |
|  | |  | mass*temperature | | 3.8 ± 4.52 | | 0.84 [12826] | | 0.4 | | mass*temperature: 0.04 [1,12826] | | 0.85 | |  |
| temperature*season | | -33156.85 | (Intercept) | | 1.42 ± 0.11 | | 12.62 [12825] | |  | |  | |  | |  |
|  | |  | temperature | | -0.12 ± 0.11 | | -1.04 [12825] | | 0.3 | | temperature: 22.38 [1,12825] | | <0.001 | |  |
|  | |  | season (winter) | | -0.45 ± 0.15 | | -3.1 [17] | | 0.01 | | season: 62.85 [2,17] | | <0.001 | |  |
|  | |  | season (spring) | | 0.65 ± 0.14 | | 4.8 [17] | | 0.001 | |  | |  | |  |
|  | |  | temperature*season (winter) | | 0.02 ± 0.14 | | 0.11 [12825] | | 0.91 | | temperature*season: 0.34 [2,12825] | | 0.71 | |  |
|  | |  | temperature*season (spring) | | 0.09 ± 0.13 | | 0.67 [12825] | | 0.5 | |  | |  | |  |
| temperature | | -33147.04 | (Intercept) | | 1.2 ± 0.04 | | 28.76 [12827] | |  | |  | |  | |  |
|  | |  | temperature | | 0.08 ± 0.04 | | 2.18 [12827] | | 0.03 | | temperature: 4.76 [1,12827] | | 0.03 | |  |
| mass*skull height | | -32791.6 | (Intercept) | | 1.43 ± 0.03 | | 42.88 [12604] | |  | |  | |  | |  |
|  | |  | mass | | 0.43 ± 0.05 | | 9.4 [15] | | 0.001 | | mass: 147.15 [1,15] | | <0.001 | |  |
|  | |  | BCH | | 0.03 ± 0.05 | | 0.55 [15] | | 0.59 | | BCH: 1.23 [1,15] | | 0.29 | |  |
|  | |  | mass*BCH | | -0.02 ± 0.08 | | -0.25 [15] | | 0.81 | | mass*BCH: 0.06 [1,15] | | 0.81 | |  |
| skull height*season | | -32783.22 | (Intercept) | | 1.3 ± 0.16 | | 8.1 [12604] | |  | |  | |  | |  |
|  | |  | BCH | | 0.01 ± 0.12 | | 0.06 [13] | | 0.95 | | BCH: 1.82 [1,13] | | 0.2 | |  |
|  | |  | season (winter) | | -0.2 ± 0.22 | | -0.91 [13] | | 0.38 | | season: 61.02 [2,13] | | <0.001 | |  |
|  | |  | season (spring) | | 0.67 ± 0.18 | | 3.73 [13] | | 0.001 | |  | |  | |  |
|  | |  | BCH*season (winter) | | 0.04 ± 0.2 | | 0.21 [13] | | 0.84 | | BCH*season: 1.34 [2,13] | | 0.3 | |  |
|  | |  | BCH*season (spring) | | -0.37 ± 0.25 | | -1.5 [13] | | 0.16 | |  | |  | |  |
| skull height | | -32733.01 | (Intercept) | | 1.39 ± 0.07 | | 20.49 [12604] | |  | |  | |  | |  |
|  | |  | BCH | | 0.04 ± 0.07 | | 0.59 [17] | | 0.57 | | BCH: 0.34 [1,17] | | 0.57 | |  |
|  | | |  | |  | |  | |  | |  | |  | |  |
| **Relative MR (VO2 ml kg^-1^ min^-1^) ~** | **AICc** | | | **Factors** | | **Estimate ± SE** | | **t-value [DF]** | | **p-value** | | **Anova LME F [DF]** | | **p-value** | |
| skull height*temperature | | 82949.02 | (Intercept) | | 157.54 ± 5.65 | | 27.88 [12221] | |  | |  | |  | |  |
|  | |  | BCH | | 9.98 ± 5.56 | | 1.8 [17] | | 0.09 | | BCH: 1.83 [1,17] | | 0.19 | |  |
|  | |  | temperature | | -5.9 ± 5.05 | | -1.17 [12221] | | 0.24 | | temperature: 1.06 [1,12221] | | 0.3 | |  |
|  | |  | BCH*temperature | | -3.23 ± 5.52 | | -0.59 [12221] | | 0.56 | | BCH*temperature: 0.34 [1,12221] | | 0.56 | |  |
| mass*skull height | | 85511.63 | (Intercept) | | 154.97 ± 4.14 | | 37.46 | |  | |  | |  | |  |
|  | |  | mass | | -1 ± 5.64 | | -0.18 [15] | | 0.86 | | mass: 0 [1,15] | | 0.95 | |  |
|  | |  | BCH | | 3.79 ± 6.21 | | 0.61 [15] | | 0.55 | | BCH: 1.21 [1,15] | | 0.29 | |  |
|  | |  | mass*BCH | | -1.64 ± 10.31 | | -0.16 [15] | | 0.88 | | mass*BCH: 0.03 [1,15] | | 0.88 | |  |
| skull height | | 85518.95 | (Intercept) | | 155.04 ± 4.04 | | 38.33 | |  | |  | |  | |  |
|  | |  | BCH | | 4.54 ± 4.05 | | 1.12 [17] | | 0.28 | | BCH: 1.26 [1,17] | | 0.28 | |  |
| skull height*season | | 85495.13 | (Intercept) | | 146.33 ± 18.96 | | 7.72 [12604] | |  | |  | |  | |  |
|  | |  | BCH | | 10.5 ± 13.9 | | 0.76 [13] | | 0.46 | | BCH: 1.17 [1,13] | | 0.3 | |  |
|  | |  | season (winter) | | 19.39 ± 25.46 | | 0.76 [13] | | 0.46 | | season: 0.15 [2,13] | | 0.86 | |  |
|  | |  | season (spring) | | 6.41 ± 21.29 | | 0.3 [13] | | 0.77 | |  | |  | |  |
|  | |  | BCH*season (winter) | | 5.37 ± 23.21 | | 0.23 [13] | | 0.82 | | BCH*season: 0.23 [2,13] | | 0.79 | |  |
|  | |  | BCH*season (spring) | | -15.97 ± 29.03 | | -0.55 [13] | | 0.59 | |  | |  | |  |
| temperature*season | | 86804.99 | (Intercept) | | 171.96 ± 13.49 | | 12.75 [12825] | |  | |  | |  | |  |
|  | |  | temperature | | -13.3 ± 13.41 | | -0.99 [12825] | | 0.32 | | temperature: 0 [1,12825] | | 1 | |  |
|  | |  | season (winter) | | -33.26 ± 17.37 | | -1.92 [17] | | 0.07 | | season: 0.8 [2,17] | | 0.47 | |  |
|  | |  | season (spring) | | -23.01 ± 15.95 | | -1.44 [17] | | 0.17 | |  | |  | |  |
|  | |  | temperature*season (winter) | | -0.52 ± 16.33 | | -0.03 [12825] | | 0.97 | | temperature*season: 1.57 [2,12825] | | 0.21 | |  |
|  | |  | temperature*season (spring) | | 19.41 ± 15.66 | | 1.24 [12825] | | 0.22 | |  | |  | |  |
| mass*temperature | | 86822.8 | (Intercept) | | 153.19 ± 4.39 | | 34.91 [12826] | |  | |  | |  | |  |
|  | |  | mass | | -1.8 ± 4.34 | | -0.41 [18] | | 0.68 | | mass: 0.11 [1,18] | | 0.74 | |  |
|  | |  | temperature | | 1.06 ± 3.98 | | 0.27 [12826] | | 0.79 | | temperature: 0.02 [1,12826] | | 0.89 | |  |
|  | |  | mass*temperature | | 3.8 ± 4.52 | | 0.84 [12826] | | 0.4 | | mass*temperature: 0.71 [1,12826] | | 0.4 | |  |
| mass*season | | 89671.3 | (Intercept) | | 159.36 ± 15.45 | | 10.31 [13262] | |  | |  | |  | |  |
|  | |  | mass | | -0.55 ± 38.34 | | -0.01 [14] | | 0.99 | | mass: 0.12 [1,14] | | 0.74 | |  |
|  | |  | season (winter) | | -15.1 ± 31.83 | | -0.47 [14] | | 0.64 | | season: 0.41 [2,14] | | 0.67 | |  |
|  | |  | season (spring) | | 4.67 ± 36.23 | | 0.13 [14] | | 0.9 | | mass*season: 0.02 [2,14] | | 0.98 | |  |
|  | |  | mass*season (winter) | | -9.68 ± 51.7 | | -0.19 [14] | | 0.85 | |  | |  | |  |
|  | |  | mass*season (spring) | | -7.41 ± 43.75 | | -0.17 [14] | | 0.87 | |  | |  | |  |
| season | | 89691.45 | (Intercept) | | 159.6 ± 7.02 | | 22.74 [13262] | |  | | season: 0.39 [2,17] | | 0.68 | |  |
|  | |  | season (winter) | | -7.35 ± 9.31 | | -0.79 [17] | | 0.44 | |  | |  | |  |
|  | |  | season (spring) | | -7.58 ± 10.12 | | -0.75 [17] | | 0.46 | |  | |  | |  |
| mass | | 89698 | (Intercept) | | 154.46 ± 3.89 | | 39.68 [13262] | |  | |  | |  | |  |
|  | |  | mass | | -1.34 ± 3.89 | | -0.35 [18] | | 0.73 | | mass: 0.12 [1,18] | | 0.73 | |  |
